# Alzheimer’s Disease: Pathological Mechanisms and Recent Insights

**DOI:** 10.2174/157015911798376181

**Published:** 2011-12

**Authors:** Dana M Niedowicz, Peter T Nelson, M. Paul Murphy

**Affiliations:** 1 Departments of Molecular and Cellular Biochemistry, University of Kentucky, Lexington, KY, USA; 2 Departments of Pathology, University of Kentucky, Lexington, KY, USA; 3 the Sanders-Brown Center on Aging, University of Kentucky, Lexington, KY, USA

**Keywords:** Amyloid, amyloid-β peptide, amyloid-β precursor protein, neurodegeneration, protein misfolding, proteinopathies, Tau.

## Abstract

Amyloidopathies cause neurodegeneration in a substantial portion of the elderly population. Improvements in long term health care have made elderly individuals a large and growing demographic group, marking these diseases as a major public health concern. Alzheimer’s Disease (AD) is the most studied form of neurodegenerative amyloidopathy. Although our understanding of AD is far from complete, several decades of research have advanced our knowledge to the point where it is conceivable that some form of disease modifying therapy may be available in the near future. These advances have been built on a strong mechanistic understanding of the disease from its underlying genetics, molecular biology and clinical pathology. Insights derived from the study of other neurodegenerative diseases, such as some forms of frontotemporal dementia, have been critical to this process. This knowledge has allowed researchers to construct animal models of the disease process that have paved the way towards the development of therapeutics. However, what was once thought to be a straightforward problem has evolved into a series of disappointing outcomes. Examination of pathways common to all neurodegenerative diseases, including the cellular mechanisms that clear misfolded proteins and their regulation, may be the best way to move forward.

## INTRODUCTION

### With Each Passing Decade, We Are Getting Older

The elderly make up a greater proportion of our population than ever before, and this proportion is increasing. Demographic trends will continue to push our collective age to higher levels for the foreseeable future [1]. Although this represents a triumph of modern medicine, it brings along with it a series of problems and challenges. One of the most serious issues that this “graying society” must confront is the burden of age-related disease. Disorders that strike late in life already consume a staggering amount of resources. Unless significant steps are taken at both national and international levels, in both the public and private sector, current signs indicate that this will become a crippling public health crisis by mid-century [2].

**Fig. (1):**
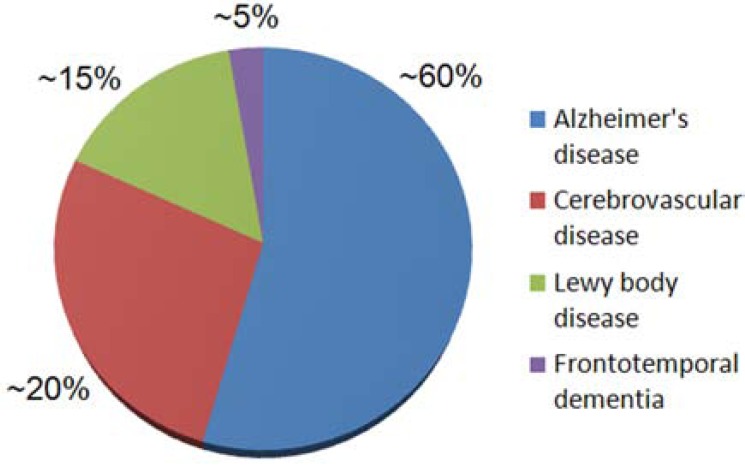
Distribution of the Major Age-Related Neurodegenerative Diseases. There are several different brain diseases that contribute substantially to cognitive impairment in the elderly. The weighted contributions shown on this pie chart are approximate and represent data collected from five different U.S. studies [8, 108-111]. Some other diseases that adversely affect cognition (including hippocampal sclerosis) affect many persons but are not discussed in this review. There is variation between studies due to population differences (*e.g.*, mean age) and methodology. Thus, we have simplified these data for illustration. Note that AD accounts for more than one-half of cognitive impairment among American aged persons. In the “oldest old” (85+), some impact from cerebrovascular pathology is almost universal, and it is normal for the brains of individuals over the age of 80 to harbor more than one type of pathology.

Neurodegenerative disease is a significant part of this problem. Although Alzheimer’s disease (AD) is the most common neurodegenerative disease (Fig. **1**), and the best known and studied, it is only one of a much larger class of age-related protein misfolding diseases that affect the central nervous system. Although there is some overlap, each disease presents with its own set of clinically distinct features and, in general, this clinical phenotype reflects the underlying neuropathology. The majority of these diseases, which we can refer to as neurodegenerative amyloidopathies, have an insidious onset, with preclinical pathology developing over many years, if not decades. Often, by the time an individual or their loved ones is aware that a problem exists, massive neurological damage has already been sustained. Since it is near certain that this damage cannot easily be repaired in the elderly brain, early detection, intervention and prevention will likely represent the future keys to successful treatment.

Each disease has one or more proteins that most likely form the core of the pathogenic process. In AD, a key player is often thought to be the amyloid-β (Aβ) peptide [3], first isolated as the principle component of amyloid deposits in the brain and cerebrovasculature of AD cases and Down’s Syndrome (DS) patients [4-6]. The second form of proteinaceous deposit in the AD brain, the neurofibrillary tangle (NFT), is comprised of polymers of a hyperpohosphorylated form of the microtubule associated protein tau, and this may be the main downstream effector of neuronal death [7]. The amount of NFTs in the cerebral cortex found upon autopsy correlates strongly with antemortem cognitive decline in clinical-pathological studies from many centers [8]. The “neuritic plaque”, which includes both the extracellular Aβ deposition and a surrounding penumbra of degenerating neurites with pathologically-folded tau, are the most specific histopathological lesions of AD. At this time AD is defined by the presence of all three of these: numerous NFTs, numerous neuritic plaques, and cognitive impairment [9]. Tau itself features prominently in additional neurodegenerative diseases, called tauopathies (a specific subgroup of the broader amyloidopathies [10]), in which it is believed to be a major factor in neurodegeneration. Tauopathies comprise frontotemporal dementia with Parkinsonism linked to chromosome 17 (FTDP17; see below), Pick’s disease, corticobasal degeneration, progressive supranuclear palsy, and others. Many types of human prion disease, such as variant Creutzfeldt-Jakob disease and Gerstmann-Straussler-Scheinker disease, exhibit amyloid deposition [11]. Deposits of the protein synuclein are commonly found in Parkinson’s disease [12] and in Lewy body dementia [13]. It has been suggested that Parkinson’s disease, Lewy body dementia and AD are all part of an overlapping disease spectrum [14]. Not all of these diseases have been examined in equal detail. However, there is much we can learn about common mechanisms involved in neurodegeneration from examining those diseases that have been extensively studied, such as AD, and this can lead to valuable insights into how these diseases might be treated.

## ALZHEIMER’S DISEASE, FRONTOTEMPORAL DEMENTIA AND THE AMYLOID CASCADE

**Fig. (2):**
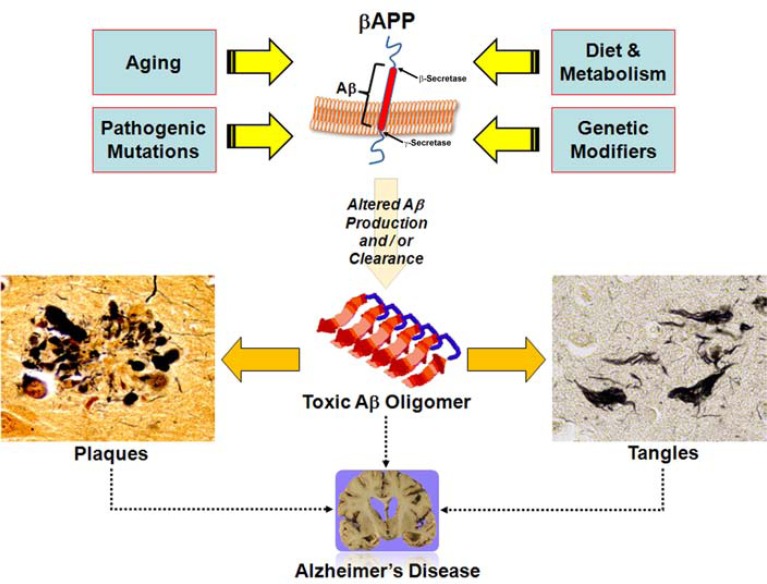
Factors Affecting Amyloid Accumulation and AD Pathology. In a broad sense, AD is characterized by neuronal degeneration, plaques and tangles. Strong evidence implicates the amyloid-β precursor protein (APP), the source of the amyloid-β peptide (Aβ), as the central player in the pathophysiology of the disease. Various factors (pathogenic mutations, genetic modifiers, diet and metabolism, and even the aging process itself) conspire so that the steady state levels of Aβ are extremely high in the AD brain. This can occur because of higher APP expression, increased APP metabolism (more β- and / or γ-secretase activity), decreased Aβ catabolism, faulty clearance of Aβ from the brain, or some combination of these processes. Elevated levels of Aβ, particularly aggregation prone species such as Aβ_42_, lead to an increase in the amount of higher order oligomeric forms of the peptide (made up of two or more Aβ molecules). These intermediates exist, at least transiently, in a toxic soluble form which probably exists both inside and outside the cell. There is evidence that oligomeric Aβ damages neurons, leads to neurofibrillary tangles and eventual cell death, and ultimately forms highly insoluble fibrils that eventually deposit as plaques in the brain parenchyma. Oligomeric Aβ also likely has other deleterious effects on neuronal function, only some of which have been characterized to date. Although AD is the best studied amyloidopathy, similar mechanisms (acting on proteins other than APP) may lie at the heart of many other neurodegenerative diseases. It is possible that knowledge of the common mechanisms contributing to these disease processes may lead to therapeutic approaches that are at least partially effective against neurodegeneration in general.

AD is the best understood neurodegenerative disease, and the most studied protein misfolding disease. The prevailing hypothesis for how AD develops is deceptively simple (Fig. **2**). The Aβ peptide is produced from the larger amyloid-β precursor protein (APP) through two sequential enzymatic activities, β- and γ-secretase. β-Secretase activity (mostly attributed to the BACE1 protein in the brain [15]) is usually thought of as the rate limiting enzyme in Aβ production. β-Secretase activity leaves a membrane bound C-terminal fragment which is then cleaved by γ-secretase. This final cleavage generates the secreted Aβ peptide and a cytosolic fragment which may offer a clue to the actual function of APP [3, 16-18]. The majority of Aβ is 40 amino acids long (Aβ_40_), but a small proportion (<10%) is slightly longer (Aβ_42_). Higher levels of Aβ_42_, the more hydrophobic and fibrillogenic form of the peptide, fosters the assembly of Aβ into progressively higher order structures, from dimers all the way up to the insoluble plaques that finally deposit in the brain [19]. Since the more soluble assembled forms of the peptide are directly toxic [20], as the total amount of Aβ increases, neurons and synapses start to suffer deleterious consequences and, ultimately, degenerate [21]. It is the widespread elimination of synapses and death of neurons that causes the crippling memory deficit that eventually leads to the inability to sustain normal daily function. This hypothesis, usually called the amyloid cascade hypothesis, is backed by an enormous amount of circumstantial evidence from multiple avenues of biological research [3].

Although the range of data is compelling, the strongest evidence for the amyloid cascade in AD is genetic. Autosomal dominant mutations that cause familial AD are present in both APP and the presenilins (PS1 and PS2). Since PS1 or PS2 form the active site of γ-secretase, this places AD causal mutations within either the substrate from which Aβ originates (APP) or within the final enzyme in the production pathway. Many mutations have now been identified (an up to date list is maintained at http://www.alzforum.org/res/com/ mut/). Mutations in the presenilins lead to a shift in the production of Aβ towards the more amyloidogenic form, generally seen as an increase in the ratio of Aβ_42_:Aβ_40_; although there is some debate on the issue, this phenomenon is usually thought to reflect a loss rather than a gain of function [22, 23]. Disease causing mutations in APP are clustered either within the Aβ region or in close proximity to the β- or γ-secretase cleavage sites. After two decades of study, the matter of the actual biological function of APP is still unresolved [24].

Even though true familial AD is rare (<5% of all cases), the overall heritability of AD is estimated at ~60% or greater, based on a study of the Swedish twin registry comprising close to 12,000 pairs of individuals with and without AD [25]. This points to the existence of multiple genetic risk factors, or modifiers, in addition to the known causative mutations. The best known of these is the apolipoprotein E (ApoE) gene on chromosome 19 [26, 27]. There are 3 common alleles of the ApoE gene: ε2, ε3 and ε4. When compared to noncarriers, individuals heterozygous or homozygous for the ε4 allele have ~3 or ~12 fold elevated risk (respectively) for developing AD in their lifetime [28]. In recent years, several other genetic risk factors, all considerably weaker than ApoE genotype, have been documented in genome wide association studies [29]. Two of the largest genome-wide association studies to date identified three genes – clusterin (also known as apolipoprotein J), phosphatidylinositol-binding clathrin assembly protein (PICALM) and complement receptor 1 (CR1) – that each contribute a small amount to the total AD risk [30, 31]. This is a continually evolving field, and an up to date database of the available evidence for each gene is freely available online (http://www.alzgene.org/TopResults.asp). Compared to the autosomal dominant mutations that cause familial AD, much less is known about how these genetic modifiers affect AD pathogenesis. One likely mechanism is that they influence the rate of Aβ aggregation [27, 29], because the ApoE4 allele certainly correlates with increased Aβ deposition in brain parenchyma and around blood vessels.

The remaining AD risk is attributable to nongenetic, or environmental, factors. A wide range of variables has been associated with altered incidence of AD. Aging is thought to be the strongest nongenetic risk factor for the disease [32]. There are multiple reasons why the general process of aging might drive the development of AD. The simplest of these is that the accumulation of amyloid pathology in the brain is a slow process that occurs over many years. Similarly, age-related decreases in normal cellular processes, such as general protein turnover, may play a major role [33]. Other systemic variables probably affect risk of AD over these long periods of time. There is compelling evidence that lifestyle, pharmaceutical use and overall health play a role in the development of the disease, although these topics have also generated controversy. For example, use of cholesterol lowering agents such as some statins [34] and some non-steroidal anti-inflammatory drugs (NSAIDs) [35] has been shown to lower AD risk in some but not all studies. A connection exists between increased risk for AD and Type 2 diabetes mellitus (T2DM), a metabolic disease that is tightly linked to obesity [36], although this is also controversial [37]. This remains an area of active research. In this vein, considerable recent attention has been given to determining the role of leptin, an adipocyte-derived peptide hormone that regulates transcription *via *multiple signal transduction pathways [38]. Low serum leptin is strongly correlated with increased AD risk, as well as greater cognitive decline during AD progression [39, 40]. Mid-life obesity is a known risk factor for AD [41]; however, T2DM and obese patients are hyperleptinemic, and yet have elevated levels of Aβ [36]. The key may lie in the fact that these individuals develop leptin resistance, and over time become insensitive to their high levels of circulating leptin [38]. In other words, leptin may act as a normal physiological brake on the amount of Aβ in the brain, and in some individuals this braking mechanism fails.

The final amount of Aβ that accumulates as amyloid deposits in the brain is determined by the interplay of several factors: how much peptide is produced (discussed briefly above), how efficiently it is degraded within the brain, and how much of it is transported out into the periphery. The enzymes neprilysin (NEP) and insulin degrading enzyme (IDE) account for most Aβ degradation [42-46], and the activity of both decrease in normal aging and in disease-affected regions [47, 48]. Neprilysin activity may also decrease in the CSF in early stages of AD [49]. In spite of these catabolic processes, a significant amount of Aβ is not degraded, and is instead transported across the blood brain barrier (BBB) into the general circulation. Soluble Aβ is exchanged across the BBB *via *the low-density lipoprotein receptor-related protein (LRP) on the abluminal (brain) side [50], and the receptor for advanced glycation end products (RAGE) on the luminal (blood) side [51]. Whether or not Aβ simply hijacks this bidirectional mechanism or if it has a specific physiological function is not known. It is possible to predict the amount of deposited amyloid in the brain if the net efflux of Aβ is known [52], and inhibiting this mechanism leads to an increase in Aβ accumulation [53]. This indicates that faulty exchange processes or, possibly, general vascular abnormalities could contribute significantly to the development of Aβ pathology in the brain [54].

There has been a persistent debate over the relative toxicity of the Aβ peptide. Over the past decade, the focus on toxicity has shifted away from largely insoluble amyloid deposits towards more soluble forms of the peptide, particularly soluble oligomeric forms of Aβ. Oligomeric forms of Aβ are orders of magnitude more cytotoxic than fibrillar Aβ (what is typically deposited in plaques), and trigger a different set of toxic events [55]. The concentration of soluble oligomers in CSF or brain interstitial fluid is in the pM range [56], and there is some evidence that insoluble Aβ deposits in the brain may serve as a reservoir for more soluble Aβ species [57]. The longer, more hydrophobic forms of Aβ oligomerize more readily than the shorter, more soluble forms [58]. There is a good correlation between the ratio of Aβ_42_ / Aβ_40_ and the age of onset in familial AD [59], and Aβ deposition *in vivo* is driven almost entirely by Aβ_42_, and not Aβ_40_ [60]. Higher order, soluble oligomeric forms of Aβ are both toxic to neurons and cause deficits in long term potentiation [20, 61], and species as small as dimers inhibit electrophysiological measures of synaptic transmission [62, 63]. Although it is widely agreed that soluble oligomers are cytotoxic, the mechanism is not yet established. It is possible that oligomers interact directly with the membrane bilayer, or through one or more cellular receptors [64, 65]. Additionally, there is also evidence that Aβ_42_ stimulates formation of neurofibrillary tangles, effectively linking the two major AD pathologies [66, 67]. Although Aβ oligomers are by far the most studied, there is good evidence that similar processes may operate in other neurodegenerative amyloidopathies [20].

**Fig. (3):**
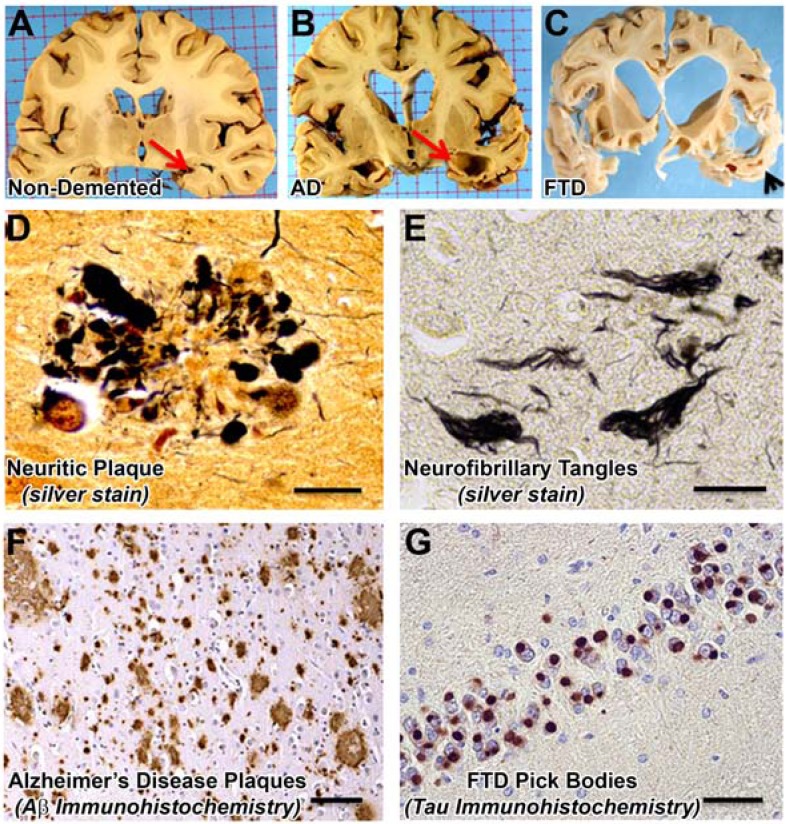
AD and FTD Pathologies. Representative gross photographs (**A**-**C**) and photomicrographs (**D**-**G**) demonstrate features of Alzheimer’s disease (AD) and frontotemporal dementia (FTD). Gross photographs of coronal slices of human brain at the level of the lateral geniculate nucleus show that AD brains can have extreme atrophy of the hippocampal formation (red arrows in A,B). A slightly more anterior slice from an FTD patient (**C**) depicts the atrophy in the region of the temporal pole (black arrowhead). Silver stains show both neuritic plaques (**D**) and neurofibrillary tangles (**E**) in AD. Immunohistochemistry is useful to stain disease-related antigens: Aβ peptide at low magnification in AD neocortex (**F**), and modified tau protein (“Pick bodies”) in the dentate granule cells in Pick’s disease, a subtype of FTD (**G**). Scale bars for photomicrographs: 20 μm in D,E; 1 mm in F, and 50 µm in G.

**Fig. (4):**
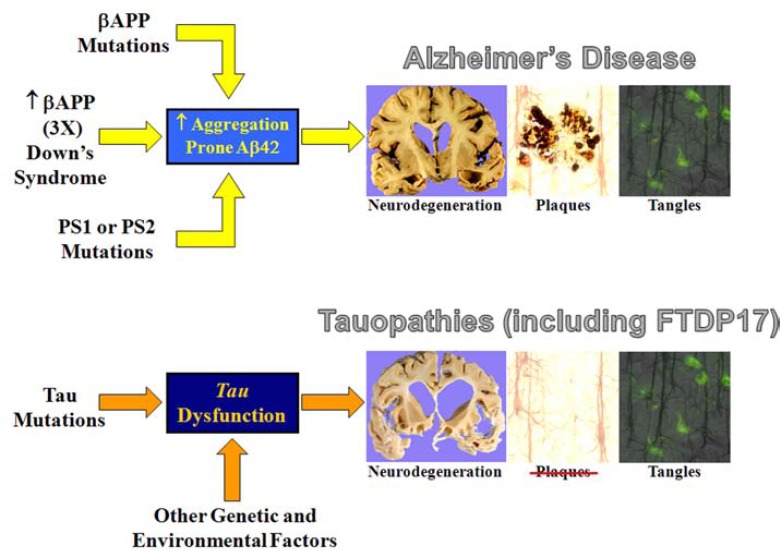
Comparison of AD and Tauopathies. In inherited or familial AD (FAD), the disease is characterized by neuronal degeneration, plaques, and tangles. FAD is caused by increased APP expression (such as in Down’s syndrome or in rare families with an extra copy of the APP gene), mutations in APP, or mutations in the presenilin genes, which all lead to an increase in aggregation- prone Aβ. In chromosome 17- linked frontotemporal dementia with parkinsonism (FTDP17), the disease is characterized by neuronal degeneration and tangles, but no plaques. In FTDP17, mutations in tau – the major component of neurofibrillary tangles – leads to tau dysfunction and / or aggregation. Other genetic and environmental factors can also lead to tauopathies. The most straight-forward conclusion is that tangle formation as a consequence of abnormalities in tau causes neurodegeneration. Since plaques are not a pathologic feature of tauopathies, we can also conclude that tau-induced neurodegeneration does not cause the formation of plaques. Since tangles occur in FAD along with Aβ pathology, a simple synthesis of what we know about both diseases indicates that Aβ (in some form) causes both tangles and neurodegeneration.

The study of non-AD forms of age-related neurodegeneration have helped expand our understanding of the underlying mechanisms that many of these diseases share. Frontotemporal dementia, or frontotemporal lobar degeneration (FTLD), has proven to be very informative. Nearly all cases of FTLD fall into 3 categories, based on the most prominent misfolded protein detected in the pathologic aggregates: tau, TDP-43 (TAR DNA binding protein 43), and Fus (fused in sarcoma) [68]. FTLD-tau disorders (Fig. **3**) have direct and obvious implications for AD. A key discovery in this area was the linkage of mutations in the *MAPT* gene (which encodes tau) on chromosome 17 to the development of a sub-type of frontotemporal dementia, FTDP17 [69, 70]. Mutations that cause FTDP17 affect tau in at least two ways [10]. First, mutations in the coding sequence may either affect the ability of tau to bind to and stabilize microtubules, or may affect its propensity towards aggregation. Second, noncoding mutations alter the ratio of splice variants, particularly leading to imbalance in the expressed microtubule binding domains. A striking characteristic of FTDP17 is that in spite of the presence of extensive tau pathology, Aβ deposits are not a feature of this disease. In contrast, genetic causes of AD (by definition) increase the amount of Aβ deposition and the development of tangle pathology. Together, these two diseases show that abnormalities in tau can lead directly to neurodegeneration, and that neurodegeneration caused by tau pathology does not cause Aβ to deposit in the brain (Fig. **4**). The corollary of this is that Aβ pathology in AD most likely occurs upstream of tau pathology.

Researchers have used knowledge of the genetic and molecular mechanisms involved in the development of AD and related diseases to generate a variety of animal models of neurodegeneration [71-73]. These models have largely been developed in mice, and have advanced to the point where some strains have pathology present at or shortly after birth [74]. Robust pathology requires the introduction of some combination of familial AD mutations into APP, PS1, or tau, with the addition of more mutations producing an acceleration of pathology. Also, although there are exceptions [75, 76], it is necessary to over express APP containing human sequence Aβ at relatively high levels (using an ectopic promoter) to drive the Aβ deposition in mice. As a consequence, in many models, the Aβ produced is present alongside a small amount of endogenous rodent Aβ, which may have some affect on the development of pathology [77-79].

Genetically modified mice have proven to be useful tools in the study of the disease process. For instance, transgenic mice expressing a form of tau mutated in FTDP17 (P301L) were used to convincingly show that Aβ controls the rate of development of tangle pathology [80, 81], demonstrating experimentally that abnormal Aβ may occur before abnormal tau in the amyloid cascade. Mouse models have also been used to study the near real-time deposition of amyloid in the brain [82]. Remarkably, a large number of potential therapeutic interventions have been shown to have some success in one or more mouse models [83-85]. It is possible that Aβ deposited in the mouse brain may be more plastic than that deposited in the human brain. This phenomenon may be related to the much shorter mouse lifespan, leading to lower levels of *in vivo* modification [86]. Less preclinical work has been carried out in models that also develop tau pathology, although this situation will likely change in the future. The absence of significant, *bona fide* tangle pathology in earlier mouse models may be one factor that accounts for why these models have turned out to be poorly predictive of clinical success (see below). For instance, tau pathology is capable of propagation within the brain and does not require the presence of Aβ pathology [87, 88]. This spread of altered tau in the brain indicates that once the disease process is triggered, the simple removal of Aβ may be insufficient because tau pathology proceeds relatively unaltered. Regardless, over the past decade, the development of AD therapeutics has been heavily reliant on mouse models, and effectiveness in these mice has become almost a necessity for proceeding to human clinical trials.

## TREATING ALZHEIMER’S DISEASE

Recently, Myriad Genetics ceased development of Tarenflurbil (Flurizan^TM^), a promising candidate treatment for early-stage Alzheimer’s disease. This was the largest phase III clinical trial for an AD drug in history, enrolling nearly 1600 patients in North America alone [89]. With the failure of Alzhemed^TM^ (tramiprosate) by Neurochem (now Bellus Health), this was the second high profile clinical failure within a year [90]. Although showing early promise as an AD therapy, Pfizer also recently announced that Lipitor® was not useful in slowing cognitive decline [91]. Add to the record the other recent therapeutic disappointments of nonsteroidal anti-inflammatory drugs (the NSAIDs Celebrex® and naproxen) [92] and estrogen replacement [93], and the tally of failure grows uncomfortably long. This streak is troubling in a number of ways. Is it enough to simply say that our first generation of disease-modifying agents are weak, and are not being administered early enough in the course of the disease, or are there other problems to consider?

Development of new therapeutics, or even new uses for old drugs, is a challenge. The odds are long, and the process near prohibitively expensive (the phase III trial of Flurizan cost in excess of $100 million dollars). Since the amyloid cascade hypothesis is supported by far more evidence than any other option, it is still the logical foundation on which to base the development of therapies that attempt to slow, or even reverse, AD. Targets include both the enzymes that generate Aβ (β- and γ-secretases), as well as the peptide itself. Because drug development of any kind is a long shot, researchers are simply placing a well-educated wager. Nevertheless, after the recent string of losses on what seemed like good bets, these high-profile clinical failures may indicate that changes may be needed in thinking about how to treat AD and other forms of neurodegenerative amyloidopathies.

All of the three recent high profile failures had decent evidence of efficacy in small early trials [94-96]. Is it really fair to say that these different approaches failed because they were on the weak side of the therapeutic spectrum? It is possible that the dosing schedules were insufficient, or the bioavailability was poor, or that any number of other pharmocodynamic variables were not optimal. Although there is likely some truth in all of these statements, they ignore potentially larger problems. For instance, all of these approaches showed significant promise in pre-clinical animal models, and the efficacy was not remarkably different. Transgenic mice over expressing mutant forms of APP have been the animal models of choice for screening potential AD therapeutics [72]. In most studies, a promising candidate treatment reduces the amount of Aβ in the brain from 25% - 75%, with the observed reduction depending on a number of factors (how it is measured, the age and strain of the mice, etc). This suggests that the relative efficacy of a given compound at removing Aβ in mice is a poor predictor of success as a treatment for AD in the clinic. Second, by extension this indicates that the animal model of choice is not as useful as was once thought.

The statement that the existing mouse models of AD are inadequate is far from heretical. It is widely appreciated that current animal models (mouse and others) only recapitulate a small portion of the human disease. In contrast to humans, APP over expressing transgenic mice never develop robust neurofibrillar pathology, nor do they show a remarkable loss of neurons. These discrepancies have been clearly recognized in the literature, but their importance is perhaps all too often largely discounted. The reasoning typically put forth for the utility of these models is based on the amyloid hypothesis: if Aβ is at the top of the amyloid cascade, the first step in the pathway to neurodegeneration, any agent that can accelerate its removal in an animal model is a good candidate for clinical trial. Based upon these key assumptions, there are still major late stage clinical trials underway – most notably immunotherapy (Elan and Wyeth, and others) and γ-secretase inhibition (Eli Lilly) – and it is possible that one of these will succeed where others have failed. However, since pre-clinical models have not yet predicted clinical success, and since these models indicated that all of these approaches were approximately equipotent (including those in current clinical trials), there does not appear to be good reason for optimism.

Many researchers have long considered anti-Aβ_42_ immunotherapy as the most promising approach geared towards clearing Aβ from the brain. Immunotherapy is one of two remaining major approaches in clinical development that is thought to have the most promise (there are many others, that are less well developed), and is perhaps the most widely studied therapeutic in the AD field. The mechanism through which immunotherapy acts is not well understood, although the balance of evidence suggests Aβ clearance through mass action or a “peripheral sink” [97]. In practice, either passive or active immunization is capable of inducing a near complete block of amyloid deposition in the brains of multiple mouse models, if administration occurs at an early age. Although later immunization is less effective, it still has some potency [98]. Immunized mice also show some cognitive improvement [99]. Two recent studies have raised what could be considered prominent warning signs. First, active immunization in a different species, aged canines, did not improve cognitive function in spite of clearing large amounts of deposited amyloid [100]. Second, a follow-up study in a small number of patients formerly enrolled in a phase I active immunotherapy study (AN1742) indicated that clinical progression and endpoint were unaltered in spite of substantial amyloid clearance [101]. The current clinical trials of anti-Aβ_42_ immunotherapy will show if these warning signs predicted the ultimate outcome, but it is reasonable to believe that they will fare no better than Flurizan, Alzhemed and Lipitor. It is in fact reasonable to expect that a future follow-up of Flurizan or Alzhemed treated patients might show a similar reduction in amyloid deposition without measurable clinical benefit.

What if immunotherapy (and γ-secretase inhibition) fails? This does not mean, as some have suggested, that the amyloid cascade model of the disease is wrong. What it does mean is that other important aspects of the disease are likely being overlooked. At least in the case of immunized patients that have come to autopsy, tau pathology appears relatively unaltered [101]. Perhaps it will turn out to be impossible to predict the success of a given approach unless it can be concurrently evaluated for efficacy against the downstream consequences of the disease, NFTs and neuronal degeneration. This will require a refocusing of development efforts using models that possess amyloid deposition along with true concurrent tangle pathology and frank neurodegeneration, rather than the surrogate markers that are frequently used. It is also possible that the end points evaluated in model systems are incorrect. Too much emphasis may have been placed on the insoluble pool of deposited amyloid rather than on the more soluble, oligomeric material. As mentioned above, the fact that many approaches can clear similar amounts of amyloid from the mouse brain could indicate that mouse amyloid is significantly more labile than the human form. Finally, perhaps more effort should be devoted towards developing models that are less reliant on the overexpression of mutant proteins. Although a complete return to the drawing board is unlikely, AD therapeutic development is clearly further away from where it once was, where it once seemed likely that a disease- modifying drug would soon be available.

There is no doubt that improving the ability of clinicians to detect AD at an early stage will help. It is possible that Aβ acts as a triggering mechanism that initiates a cascade of tau pathology and neurodegeneration early in the disease process and, after the initial burst of amyloid pathology, neurodegeneration advances whether or not Aβ is present. Recent studies show that tau pathology can progress and propagate between cells, and it is possible that this mechanism might be at work in the human disease [87, 88]. This scenario is akin to a runaway train, in that once the initial momentum (Aβ) is imparted to the process, it may be difficult or impossible to halt if this factor is removed. In this situation, the only hope is intervention in the cascade at the earliest possible moment. Based on current conceptualizations of AD, this would likely represent a phase with no or minimal hints of incipient dementia, since even mild cognitive impairment usually presents with both amyloid and tangle pathology [102]. It is widely hoped that advances in amyloid imaging and the use of more refined biomarkers of the disease will be the key [103]. Unfortunately, the reliable diagnosis of AD at the presymptomatic stage will be a costly undertaking, and is unlikely to occur in the near future. This makes the prospects for amyloid lowering agents as an AD monotherapy uncertain, at least for the time being.

Combining an Aβ lowering agent with a tau clearance strategy may be what is called for, but it is by no means the only possibility. Neurodegenerative amyloidopathies, since they all involve protein misfolding to some degree, will all share some pathologic features in common. A worthwhile treatment possibility might involve targeting one or more of these common pathways. This could have the added advantage of being efficacious for the treatment of multiple diseases, most of which have too small a patient population to spur more traditional large scale drug development efforts. There are many common biological mechanisms that are perturbed in neurodegenerative disease. As just one example, the process of autophagy is triggered across a range of neurodegenerative diseases [104]. This is likely a protective response to clear large quantities of aggregated protein. In fact, activating autophagy can both extend lifespan [105] and reverse amyloid pathology in mice [106]. Activation of a general clearance mechanism coupled with the inhibition of processes that generate the specific amyloidogenic protein may evolve into a promising general treatment strategy for neurodegenerative disease [107].

## CONCLUSION

Although much is now known about Alzheimer’s Disease and related neurodegenerative conditions, understanding is far from complete. After several high profile failures based almost entirely on the removal of Aβ, it is unclear how many additional drug trials will move forward based solely on the assumption that clearing this peptide will be an effective means of treating the disease. This comes at a time when the number of individuals with AD is climbing and the human and economic costs are growing at a staggering rate. Although it could be argued that some recent therapeutic failures might work well as preventative agents if given earlier, it is unlikely that there will be lengthy and expensive primary prevention trials for drugs that have already been branded as clinical disappointments. However, if a point is reached where the remaining major clinical trials also fail, AD therapeutics will be entering into unsettled scientific territory. Future progress will face a far greater challenge than simple model tweaking, one that will require a reconsideration of the most basic assumptions of the past two decades.
